# The Pattern of Metastatic Breast Cancer: A Prospective Head-to-Head Comparison of [^18^F]FDG-PET/CT and CE-CT

**DOI:** 10.3390/jimaging9100222

**Published:** 2023-10-12

**Authors:** Rosa Gram-Nielsen, Ivar Yannick Christensen, Mohammad Naghavi-Behzad, Sara Elisabeth Dahlsgaard-Wallenius, Nick Møldrup Jakobsen, Oke Gerke, Jeanette Dupont Jensen, Marianne Ewertz, Malene Grubbe Hildebrandt, Marianne Vogsen

**Affiliations:** 1 Department of Nuclear Medicine, Odense University Hospital, DK-5000 Odense, Denmark; rosa.gram-nielsen@regionh.dk (R.G.-N.); mnb91@rsyd.dk (M.N.-B.); sara.elisabeth.wallenius@rsyd.dk (S.E.D.-W.); nick.uhrbrand.moeller.jakobsen@rsyd.dk (N.M.J.); malene.grubbe.hildebrandt@rsyd.dk (M.G.H.); 2 Department of Clinical Research, University of Southern Denmark, DK-5000 Odense, Denmark; mewertz@health.sdu.dk; 3 Department of Radiology, Odense University Hospital, DK-5000 Odense, Denmark; ivar.yannick.christensen@rsyd.dk; 4 Centre for Personalized Response Monitoring in Oncology (PREMIO), Odense University Hospital, DK-5000 Odense, Denmark; 5 Department of Nuclear Medicine, University Hospital of Southern Denmark, DK-7100 Vejle, Denmark; 6 Department of Oncology, Odense University Hospital, DK-5000 Odense, Denmark; jeanette.dupont.roenlev@rsyd.dk; 7 Centre for Innovative Medical Technology, Odense University Hospital, DK-5000 Odense, Denmark; 8 Odense Patient Data Explorative Network, Odense University Hospital, DK-5000 Odense, Denmark

**Keywords:** [^18^F]FDG-PET/CT, CE-CT, metastatic breast cancer, the proportion of agreement, metastatic distribution

## Abstract

The study aimed to compare the metastatic pattern of breast cancer and the intermodality proportion of agreement between [^18^F]FDG-PET/CT and CE-CT. Women with metastatic breast cancer (MBC) were enrolled prospectively and underwent a combined [^18^F]FDG-PET/CT and CE-CT scan to diagnose MBC. Experienced nuclear medicine and radiology physicians evaluated the scans blinded to the opposite scan results. Descriptive statistics were applied, and the intermodality proportion of agreement was used to compare [^18^F]FDG-PET/CT and CE-CT. In total, 76 women with verified MBC were enrolled in the study. The reported number of site-specific metastases for [^18^F]FDG-PET/CT vs. CE-CT was 53 (69.7%) vs. 44 (57.9%) for bone lesions, 31 (40.8%) vs. 43 (56.6%) for lung lesions, and 16 (21.1%) vs. 23 (30.3%) for liver lesions, respectively. The proportion of agreement between imaging modalities was 76.3% (95% CI 65.2–85.3) for bone lesions; 82.9% (95% CI 72.5–90.6) for liver lesions; 57.9% (95% CI 46.0–69.1) for lung lesions; and 59.2% (95% CI 47.3–70.4) for lymph nodes. In conclusion, bone and distant lymph node metastases were reported more often by [^18^F]FDG-PET/CT than CE-CT, while liver and lung metastases were reported more often by CE-CT than [^18^F]FDG-PET/CT. Agreement between scans was highest for bone and liver lesions and lowest for lymph node metastases.

## 1. Introduction

Metastatic breast cancer (MBC) is considered an incurable disease with a 5-year overall survival of only 25% [1,2,3]. An accurate diagnostic workup is paramount for staging and monitoring treatment effects [4]. Various imaging modalities have been suggested for diagnosing MBC; however, contrast-enhanced computed tomography (CE-CT) and bone scintigraphy are often used in clinical practice.

[^18^F]-fluorodeoxyglucose-positron emission tomography/computed tomography ([^18^F]FDG-PET/CT) has shown superior accuracy compared with conventional imaging for diagnosing distant metastases [5,6,7,8,9,10]. Specifically, it has been suggested that [^18^F]FDG-PET/CT has a higher sensitivity when assessing metastatic bone lesions and higher specificity for metastatic liver lesions than CE-CT [6,11,12]. Consequently, [^18^F]FDG-PET/CT has been added as a potential replacement for conventional imaging in recent international guidelines [4,13].

Accurate imaging is essential before initiating medical treatment for MBC, as the treatment response assessment depends on the disease burden at baseline. Detecting bone metastases also implies the initiation of bone-modifying agents that can positively impact morbidity, quality of life, and survival [14,15]. Further, imaging may have clinical implications for patients suspected of oligometastatic disease (OMD), as local therapy has been suggested to improve outcomes through individualized treatment strategies [16,17,18].

Since [^18^F]FDG-PET/CT has recently been recognized in international guidelines for breast cancer staging, it may lead to a broader implementation of [^18^F]FDG-PET/CT. Ultimately, [^18^F]FDG-PET/CT could provide a different perception of the metastatic pattern in MBC compared to what we know from conventional imaging.

To our knowledge, no previous studies have compared the agreement between [^18^F]FDG-PET/CT and CE-CT to assess the metastatic pattern in patients with MBC. Therefore, this prospective study aimed to compare the distribution of metastasis in women with MBC when assessed by [^18^F]FDG-PET/CT and CE-CT, respectively. The objectives were to compare lesion-based and organ-specific numbers of metastasis and the intermodality proportion of agreement for [^18^F]FDG-PET/CT and CE-CT.

## 2. Materials and Methods

This prospective agreement study was conducted at Odense University Hospital, Denmark, between September 2017 and August 2019. The Guidelines for Reporting Reliability and Agreement Studies (GRRAS) were used to report the study results [19].

Enrolled patients were identified from previous studies. Baseline scans for patients with newly diagnosed MBC from a previous study (NCT03358589) were eligible for analysis. Diagnostic accuracy and response monitoring results have been published elsewhere [8,20,21,22].

### 2.1. Patients

Eligible patients were more than 18 years of age, had signed a written consent statement, and were biopsy-verified with either de novo or recurrent MBC. Patients were excluded if they were undergoing treatment for other invasive cancers at the time of inclusion, were pregnant, or had conditions that prevented the patients’ comprehension of the study’s conduct.

The collected data consisted of patient- and disease-specific characteristics, age at MBC diagnosis, pathology reports, medical reports, [^18^F]FDG-PET/CT scan reports, and CE-CT scan reports. Data were stored and managed in secure systems such as REDCap (Research Electronic Data Capture, v. 13.7.15, Vanderbilt University, Nashville, TN, USA) and SharePoint (Microsoft, Seattle, WA, USA).

### 2.2. Image Technique

An [^18^F]FDG-PET/CT scan combined with CE-CT was performed simultaneously for each patient, in which the FDG-PET with low-dose CT was performed first, followed by the CE-CT scans. The PET imaging was conducted from the top of the skull to the mid-thigh approximately 60 ± 5 min after the intravenous administration of 4 MBq [^18^F]FDG per kilogram of body weight. Routine monitoring of blood sugar levels was performed, and patients fasted for a minimum of four hours before the [^18^F]FDG injection. All scans were conducted using PET/CT scanners, namely the GE Discovery MI 4- or 5-ring PET/CT (GE Healthcare, Buckinghamshire, UK), adhering to established guidelines from the European Association of Nuclear Medicine [23]. First, a helical CT-scan was acquired with or without ultravist contrast (ultravist 370 mg/mL at 0.8 mL/kg bodyweight) following a CT protocol with a scan field-of-view (FOV) of 70 cm, tube voltage of 120 kVp, pitch = 0.984, and GE automatic exposure control (GE smartmA, ranging from 80 to 400 mA, with NI fixed at 25). This was followed by a TOF PET scan with a bed overlap of 40% and an acquisition time of 9 min per bed on the 4-ring scanners and 6 min per bed on the 5-ring scanners.

PET data sets were subject to reconstruction in a display field of view of 70 cm using two distinct techniques: time-of-flight 3D ordered subset expectation maximization (OSEM) (GE VPFX, with 4 iterations and 17 subsets) incorporating point-spread-function correction (GE SharpIR), as well as Q.Clear (with β = 250), employing matrix sizes of either 128 × 128 or 256 × 256 (pixel size 2.74 × 2.74 mm). Iterative processing encompassed corrections for attenuation, scatter, randoms, deadtime, and normalization. Attenuation correction was predicated on the preceding diagnostic helical scan.

Diagnostic CE-CT scans were obtained using a range of scanners, including the GE VCT, GE VCT XT, GE HD 750HD, Siemens Somatom Definition Flash, or Siemens Somatom Force (Siemens Healthineers, Munich, Germany). The settings for the GE scanners encompassed a tube voltage of 120 kV and Smart mA settings in the range of 100–750 mA, with Auto mA functionality, a rotation time of 0.5 s, a pitch of 0.984:1, and a Noise Index ranging from 40 to 47, contingent upon the specific scanner type (either HD 750 or VCT) due to detector specifications. The ASiR level was set at 40%, with detector coverage of 40 mm. Three reconstruction modes were employed, including soft (for 0.625 mm axial slices and 5 mm coronal and sagittal slices), Standard, and Lung (reconstructed in 5 mm axial slices). For Siemens Flash scanners, the settings comprised a tube voltage of 120 kV, reference mAs of 150, a rotation time of 0.5 s, and a pitch of 0.9. Detector coverage was 40 mm, utilizing 0.6 mm × 128 detectors, with SAFIRE level set at 3. For Siemens Force scanners, the settings included a tube voltage of 120 kV, reference mAs of 110, a rotation time of 0.5 s, and a pitch of 0.6. Detector coverage was 60 mm, employing 0.6 mm × 192 detectors, with the AD-MIRE level set at 2. Reconstructions for the Flash scanner utilized I31f medium Smooth and I50f medium Sharp ASA kernels, with I31f for 0.625 mm and 5 mm axial, coronal, and sagittal slices, while Lung window reconstruction utilized I50f medium Sharp ASA in 5 mm axial slices. Reconstructions for the Force scanner were performed using Br40 and Bl57 kernels, with Br40 for 0.625 mm and 5 mm axial, coronal, and sagittal slices, and Lung window reconstruction utilizing Bl57 in 5 mm axial slices [24].

### 2.3. Biopsies

Patients were included in the study if MBC was confirmed by biopsy. Patients with de novo MBC were confirmed by biopsies from the primary tumor and a metastatic lesion in cases of oligometastatic disease or clinical doubt about the diagnosis as described in the previously reported accuracy study [8]. Patients with recurrent MBC were all verified by biopsy from a suitable metastatic lesion, but not all metastatic lesions could be confirmed [20]. Biopsies underwent standard pathology examination, including immunohistochemistry for estrogen receptor (ER) and human epidermal growth receptor 2 (HER2) status.

### 2.4. Image Interpretation

The [^18^F]FDG-PET/CT and CE-CT scans were assessed prospectively in daily clinical practice with confirmatory biopsies from metastatic lesions.

Experienced nuclear medicine and radiology physicians evaluated the scans blinded to the opposite scan results. For research purposes, the [^18^F]FDG-PET/CT scans were evaluated prospectively after the assessment in daily routine, meaning that several nuclear medicine specialists evaluated these scans. The CE-CT scans were evaluated retrospectively by a single radiologist. Both physicians graded their findings once on a 5-point Likert scale based on suspicion of malignancy of the lesions: 0—“no suspected metastatic lesions”; 1—“assumingly no metastatic lesions”; 2—“lesions could be as benign as malignant”; 3—“suspected metastatic lesions”; and 4—“highly suspected metastatic lesions” were present.

We defined OMD as 1–4 metastatic lesions. The assessment included the organ-specific number of metastatic lesions. OMD was defined as a maximum number of 4 metastatic lesions across all organs [17].

### 2.5. Statistical Analysis

Descriptive statistics were used to describe patient characteristics and the number of metastatic lesions in each organ. The data were assessed according to the data type with medians, ranges, and frequencies. The data for the visual assessment were dichotomized into non-metastatic for Likert 0–1 and metastatic for Likert ≥ 2.

Agreement analyses were based on the lesion-based proportion of agreement between [^18^F]FDG-PET/CT and CE-CT. Cohen’s kappa (κ) was used to evaluate the strength of intermodality agreement between [^18^F]FDG-PET/CT and CE-CT for metastatic lesions and was categorized as follows: κ < 0.00 as ‘poor’ agreement, κ: 0.00–0.20 as ‘slight’ agreement, κ: 0.21–0.40 as ‘fair’ agreement, κ: 0.41–0.60 as ‘moderate’ agreement, κ: 0.61–0.80 as ‘substantial’ agreement, and κ: 0.81–1.00 as ‘almost perfect’ agreement [25]. The proportion of agreement and κ were supplemented by 95% confidence intervals (95% CI). Data were analyzed using Stata/IC 17.0. (StataCorp, College Station, TX 77845, USA)

## 3. Results

Twenty-four patients with de novo MBC and 52 patients with recurrent MBC were combined into a cohort of 76 patients with biopsy-verified MBC.

Most patients had estrogen receptor (ER)-positive disease (49/76, 64.5%) and had the biopsy taken from a metastatic bone lesion (23/76, 30.3%). Most patients diagnosed with recurrent metastatic breast cancer received prior adjuvant treatment (37/52, 71.2%) (Table 1).

### 3.1. The Distribution of Metastases

**Figure 1:**
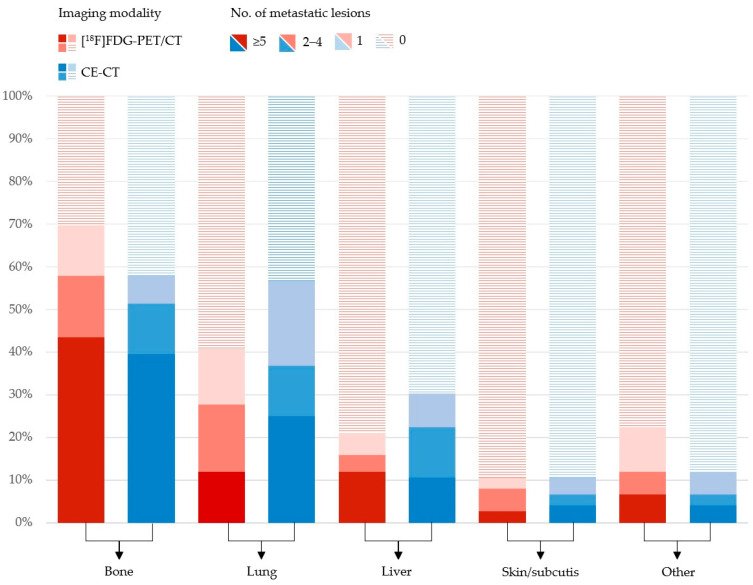
Distribution of organ-specific numbers of metastatic lesions in 76 patients with metastatic breast cancer rated by [^18^F]FDG-PET/CT (red) and CE-CT (blue).

**Figure 2:**
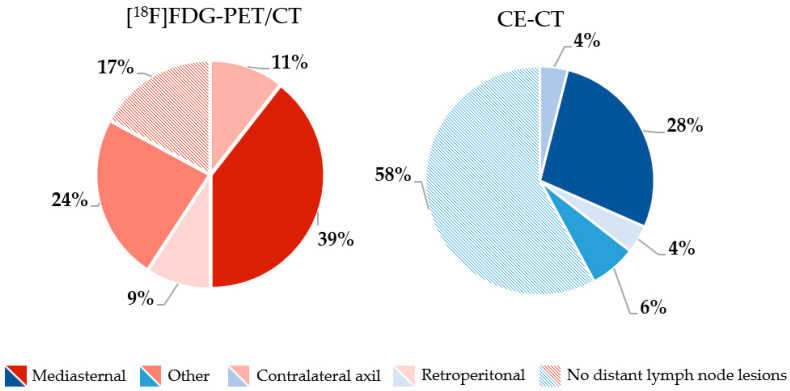
Frequency (%) of locations of distant lymph node metastases rated by [^18^F]FDG-PET/CT (red) and CE-CT (blue).

More bone metastases were detected by [^18^F]FDG-PET/CT compared with CE-CT 53/76 (69.7%) vs. 44/76 (57.9%), whereas CE-CT detected more lung metastases and liver metastases than [^18^F]FDG-PET/CT 31/76 (40.8%) vs. 43/76 (56.6%) and 16/76 (21.1%) vs. 23/76 (30.3%). Further organ-specific numbers of metastatic lesions are shown in Figure 1. In general, more metastatic lesions in other organs were detected by [^18^F]FDG-PET/CT, including distant lymph nodes. In the latter, mediastinal lymph nodes were the most common site for metastatic spread (Figure 2).

### 3.2. Intermodality Agreement

Cross-tabulations of [^18^F]FDG-PET/CT and CE-CT for the assessment of the number of metastatic lesions are seen in Table 2. The overall intermodality proportion of agreement between [^18^F]FDG-PET/CT and CE-CT for diagnosing metastatic lesions was 71.1% (95% CI 59.5–80.9) when the lesions located in lymph nodes were excluded. The highest proportion of agreement was found in the detection of liver metastases (κ-value 0.62, 95% CI 0.48–0.77), leaving the level of agreement substantial. In contrast, only fair agreement was found in detecting lung metastases, with a κ-value of 0.37 (95% CI 0.24–0.50). The proportion of agreement for detecting lymph node metastases is listed in Table 3.

**Figure 3:**
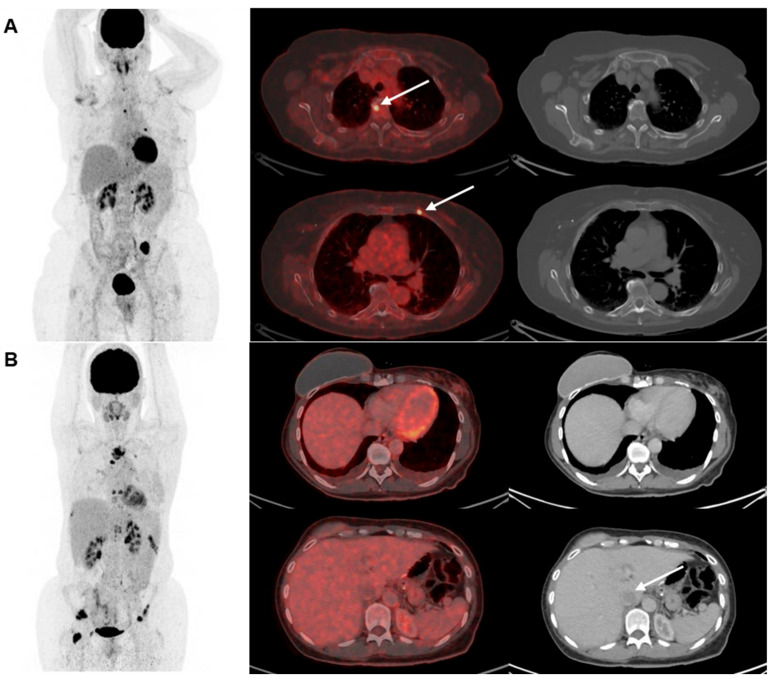
Two examples of disagreement between [^18^F]FDG-PET/CT and CE-CT for detecting metastatic lesions in (**A**) bone and (**B**) liver. Maximum intention projection images are shown in the left column, cross-sectional fused [^18^F]FDG-PET/CT images in the middle row, and CE-CT images in the right row. (**A**) shows a 67-year-old woman with biopsy-verified bone metastases where [^18^F]FDG-PET/CT detected >5 lesions with focally increased [^18^F]FDG-uptake (two illustrated by the arrows) compared with CE-CT suggesting only one lesion. (**B**) shows a 46-year-old woman with metastasis verified with a bone biopsy. CE-CT suggested 2–4 metastases in the liver (one illustrated by an arrow), while [^18^F]FDG-PET/CT did not suggest liver metastases.

Two examples of disagreement between [^18^F]FDG-PET/CT and CE-CT for detecting metastatic lesions in bone and liver leasions are seen in Figure 3.

### 3.3. Oligometastatic Disease

OMD was assessed in 19 (25%) vs. 15 (20%) patients by [^18^F]FDG-PET/CT and CE-CT, respectively. [^18^F]FDG-PET/CT assessed one lesion in 8 (10.5%) patients and 2–4 lesions in 11 (14.5%) patients, while CE-CT assessed one lesion in 7 (9.2%) patients and 2–4 lesions in 8 (10.5%) patients.

In seven cases, the two modalities agreed on a limited disease burden. Most patients (46/76, 61%) had more than five metastatic lesions according to [^18^F]FDG-PET/CT and CE-CT. One patient had metastases solely in lymph nodes (Table 2).

## 4. Discussion

In this prospective study of intermodality agreement, more bone and lymph node metastases were detected by [^18^F]FDG-PET/CT than CE-CT. In contrast, more metastases to the lung and liver were detected by CE-CT, resulting in only a fair overall agreement between the two modalities.

The strength of this study was the prospective design with patient enrollment from daily clinical practice and confirmatory biopsies of metastatic lesions. Further, experienced nuclear medicine and radiology specialists assessed the [^18^F]FDG-PET/CT and CE-CT scans.

Limitations include the single-center setup and relatively small sample size, which restrict the generalization of the results. As the CE-CT scans were assessed retrospectively, the radiologist had access to bookmarks and subsequent scans. This may have impacted the evaluation of CE-CT scans and provided the radiologist with knowledge of some lesions beforehand. [^18^F]FDG-PET/CT scans were evaluated prospectively in the daily routine, meaning that several nuclear medicine specialists evaluated these scans, while all CE-CT scans were evaluated retrospectively by a single radiologist. These physicians performed their grading once; therefore, we cannot perform interrater assessments. The motivation for assessing the [^18^F]FDG-PET/CT prospectively in daily routine was to mimic daily routine and, thereby, contribute to increasing externally valid results. We acknowledge, though, that we cannot account for the potential rater variability in grading assessments. The lack of a reference standard to verify the number of suspected metastases is another limitation. Follow-up was not used to confirm the presence of metastasis since metastatic lesions can resolve for both benign reasons and due to response to treatment. As most patients had ER+ disease with long progression-free survivals, confirmation by progression of lesions was not feasible. In this study, different reconstruction techniques were used for the PET images. Specifically, both OSEM and Q. Clear reconstruction methods were employed. While both methods have demonstrated nearly identical clinical accuracy [26], it could have been better to use the Q.Clear reconstruction algorithm in the evaluation of all scans with the potential for smaller lesion detection [27]. Finally, the research objectives differ from the lesion-based objectives listed on ClinicalTrials.gov due to the lack of a proper reference standard for all lesions.

No other studies have, to our knowledge, compared the lesion-based proportion of agreement between [^18^F]FDG-PET/CT and CE-CT; however, previous accuracy studies in breast cancer have found [^18^F]FDG-PET/CT to have a higher sensitivity for detecting bone and distant lymph node metastases compared with CE-CT [6,28,29], which is in line with our findings. Detecting bone metastases impacts treatment decisions by adding bone-modifying agents that can positively impact morbidity, quality of life, and survival [14,15]. The differences in sensitivity and specificity in detecting metastatic lesions between [^18^F]FDG-PET/CT and CE-CT reduce the agreement between the two imaging modalities at different metastatic sites.

Histopathological and biological factors can affect the degree of [^18^F]FDG uptake in metastatic lesions, with lower [^18^F]FDG uptake in metastatic lesions from lobular carcinomas and low-grade tumors and an increased risk of false-negative assessment of [^18^F]FDG-PET/CT [30,31]. [^18^F]Flouroestradiol (FES) and fibroblast activation protein inhibitors (FAPI) might be upcoming tracers to overcome these limitations of [^18^F]FDG-PET/CT [32,33,34,35].

Diagnosing metastases by CE-CT depends on anatomical changes in size and morphology, which may make it difficult to discern the difference between malignant and benign anatomy [7]. CE-CT has a lower specificity for the detection of metastatic lesions in the liver [6,36], which may explain why we found more liver metastases by CE-CT than by [^18^F]FDG-PET/CT. Detection of liver metastasis has profound prognostic consequences for the patient, and it is crucial for clinical management and later response monitoring to have a reliable visualization of the burden of the disease before initiating treatment. Metastatic lesions in the lung were also assessed more often by CE-CT; however, the true origin of such lesions can be hard to determine. The lung lesions could also originate from benign nodules or primary lung cancer and will often need follow-up [37]. Unfortunately, due to this study design and conduct, we cannot assess which imaging modality is more accurate than the other.

The concept of OMD was first introduced as a metastatic disease limited in its spread, and affected patients could potentially be amenable to metastasis-directed therapy [38]. The consensus on the definition of OMD in recent research seems to be that OMD consists of a maximum of ≤5 metastatic lesions; however, with great variation in the extent of disease spread and status [17]. Recent research suggests that metastasis-directed therapy can be clinically favorable when treating OMD, especially regarding local disease control and the few adverse effects connected to the treatment [18,39,40,41]. Such treatments include surgery, radiotherapy, or ablative techniques; however, the success of metastasis-directed therapy depends on imaging tools with high sensitivity, specificity, and accuracy [18,38]. In our study [^18^F]FDG-PET/CT and CE-CT often disagreed about OMD. Hence, in 11 patients, one of the imaging modalities assessed OMD, while the other modality assessed more than five metastatic lesions. In these cases, we could not confirm which imaging modality had the correct assessment due to a lack of reference standards for lesion-based analysis. A newer randomized trial compared the addition of metastasis-directed therapy with standard systemic therapy in patients with oligometastatic breast cancer. This study failed to show any improvement in progression-free or overall survival with the addition of metastasis-directed therapy [42]; however, the efficacy of metastasis-directed therapy in MBC needs further research.

In addition to the comparative analysis of [^18^F]FDG-PET/CT and CE-CT, it is worth acknowledging the evolving landscape of automated segmentation methods in multimodal oncology imaging. A recent study has showcased the potential of fully automatic approaches for segmentation, particularly in the context of combining PET and MR images for treatment planning. These advanced methods enable the integration of anatomical and metabolic information, enhancing the delineation of ROIs such as lesions [43]. Similarly, the study by Baek et al. highlights the power of deep segmentation networks in predicting patient survival based on tumor segmentation from PET-CT images [44]. These networks, trained to perform tumor segmentation tasks, reveal a rich set of survival-related image features, offering valuable prognostic insights. While our current study primarily focuses on the comparative analysis of [^18^F]FDG-PET/CT and CE-CT in breast cancer metastasis detection, the advancements in automated segmentation techniques underscore the potential for more precise and informative ROI delineation in multimodal oncology imaging, which may have implications for future research and clinical practice [45,46].

From a patient’s perspective, the two modalities have different pros and cons. On the one hand, [^18^F]FDG-PET/CT seems to be a more reliable tool for detecting distant metastasis than CE-CT [6,47]. Still, on the other hand, it requires longer examination time and the potential risk of detecting incidental findings, for which patients should undergo potentially unnecessary examinations [8,20,48]. CE-CT is cheaper and widely available; however, for bone metastasis detection, an additional bone scan is recommended in international guidelines [4,13]. Bone scans (magnetic resonance imaging (MRI), fluorine 18–Sodium Fluoride (18F–NaF) PET/CT, or bone scintigraphy) often require patient appearance on separate days, and not all patients are fit for MRI, especially due to claustrophobia. These pros and cons must be weighed when choosing the optimal diagnostic modality for detecting distant metastases from breast cancer.

Recent advancements in medical imaging, particularly the integration of artificial intelligence (AI), offer promising prospects for enhancing the diagnostic performance of both [^18^F]FDG-PET/CT and CE-CT. AI-driven algorithms have shown the potential to improve the accuracy of diagnosing metastatic spread, especially in scenarios like axillary lymph node metastasis in breast cancer [49,50]. These AI models, powered by deep learning techniques, can aid clinicians in making more precise and efficient diagnostic decisions, potentially reducing the need for unnecessary invasive procedures. The integration of AI into the interpretation of imaging studies is an evolving frontier that may further refine the diagnostic capabilities of molecular-based imaging in the future. Furthermore, these AI-driven networks not only facilitate accurate and efficient lesion identification but also provide valuable imaging biomarkers. Many biomarkers (SULpeak, TLG, PBI, and PLI) have demonstrated their potential in assessing treatment response. Notably, SUL_peak_ exhibited a significant decrease between baseline and follow-up scans, underscoring its diagnostic accuracy in evaluating patients’ responses to treatment. These networks represent a significant step toward enhanced diagnosis and monitoring. Nonetheless, further investigations are essential to explore the prognostic value of each imaging biomarker for predicting overall survival and progression-free survival [51].

We encourage more studies comparing [^18^F]FDG-PET/CT to conventional imaging modalities for the diagnosis of metastatic lesions in breast cancer, as it could guide treatment development and decisions. Preferably, such studies should include the role of artificial intelligence in improving the diagnostic performance of [^18^F]FDG-PET/CT.

## 5. Conclusions

Bone and distant lymph node metastases were reported more often by [^18^F]FDG-PET/CT than CE-CT, while liver and lung metastases were reported more often by CE-CT. The agreements between scans were highest for bone and liver lesions and lowest for lymph node metastases. These findings may impact treatment decisions, and the choice of diagnostic modality should be considered when staging and planning treatment for MBC patients.

## Figures and Tables

**Table 1:** Characteristics of 76 included women with biopsy-verified metastatic breast cancer.

Characteristics	Number
Age (year)	
Median (range)	71.2 (38.1–91.1)
Site of biopsy, N (%)	
Bone	23 (30.3)
Liver	10 (13.2)
Lung	2 (2.63)
Brain	1 (1.32)
Skin	2 (2.63)
Lymph node	14 (18.4)
Pleural fluid	3 (3.95)
Other	21 (27.7)
ER ^1^-status from biopsy, N (%)	
Positive 1–100%	49 (64.5)
Negative	7 (9.21)
Unknown	20 (26.3)
HER2 ^2^-status from biopsy, N (%)	
Positive	9 (11.8)
Negative	58 (76.32)
Unknown	9 (11.8)
Adjuvant treatment ^3^, N (%)	
Neoadjuvant +/− adjuvant	4 (7.69)
Adjuvant	37 (71.2)
No medical treatment	11 (21.2)

^1^ ER: Estrogen receptor. ^2^ Human epidermal growth factor 2. ^3^ Women with recurrent metastatic breast cancer (*n* = 52).

**Table 2:** Cross tabulations and agreement between [^18^F]FDG-PET/CT and CE-CT for the visual assessment of the number of metastatic lesions in all metastatic sites, bone, liver, and lung. Bold numbers indicate perfect agreement between the two modalities.

	CE-CT				
[^18^F]FDG-PET/CT	Number of Lesions				
	0	1	2–4	≥5	Total
**All lesions** ^1^					
0	**1** ^2^	4	0	1	6
1	1	**2**	1	4	8
2–4	2	0	**5**	4	11
≥5	2	1	2	**46**	51
Total	6	7	8	55	**76**
Agreement (95% CI)	Expected agreement	Kappa, κ (95% CI)	Std. Err.	Z	Prob > Z
71.1% (59.5–80.9)	51.7%	0.40 (0.29–0.59)	0.075	5.35	0.00
**Bone lesions**					
0	**22**	0	1	0	23
1	6	**2**	0	1	9
2–4	3	1	**6**	1	11
≥5	1	2	2	**28**	33
Total	32	5	9	30	**76**
Agreement (95% CI)	Expected agreement	Kappa, κ (95% CI)	Std. Err.	Z	Prob > Z
76.3% (65.2–85.3)	32.4%	0.65 (0.51–0.79)	0.074	8.83	0.00
**Lung lesions**					
0	**30**	11	2	2	45
1	1	**3**	5	1	10
2–4	2	1	**2**	7	12
≥5	0	0	0	**9**	9
Total	33	15	9	19	**76**
Agreement (95% CI)	Expected agreement	Kappa, κ (95% CI)	Std. Err.	Z	Prob > Z
57.9% (46.0–69.1)	33.1%	0.37 (0.24–0.50)	0.067	5.52	0.00
**Liver lesions**					
0	**52**	3	4	1	60
1	0	**2**	2	0	4
2–4	1	0	**2**	0	3
≥5	0	1	1	**7**	9
Total	53	6	9	8	**76**
Agreement (95% CI)	Expected agreement	Kappa, κ (95% CI)	Std. Err.	Z	Prob > Z
82.9% (72.5–90.6)	57.2%	0.60 (0.46–0.75)	0.074	8.12	0.00

^1^ Includes reported bone, liver, lung, subcutis, skin, brain, and other lesions for [^18^F]FDG-PET/CT and CE-CT. ^2^ This patient had metastases only in the lymph nodes that were not included in the agreement analysis for lesions in organs.

**Table 3:** Cross tabulations and agreement between [^18^F]FDG-PET/CT and CE-CT for the visual assessment of lymph node metastases were ranked on a 5-point Likert scale. Bold numbers indicate perfect agreement between the two modalities.

	CE-CT					
FDG-PET/CT	Likert Scale					
	0	1	2	3	4	Total
**Lymph nodes**						
0	**29**	0	1	1	0	31
1	0	**0**	0	0	0	0
2	1	0	**0**	2	0	3
3	5	0	2	**1**	0	8
4	13	0	2	4	**15**	34
Total	48	0	5	8	15	**76**
Agreement (95% CI)	Expected agreement	Kappa, κ (95% CI)	Std. Err.	Z	Prob > Z	
59.2% (47.3–70.4)	36.0%	0.36 (0.22–0.50)	0.072	5.04	0.00	

## Data Availability

The data that supports the findings of this study is available from the corresponding author upon reasonable request.
